# Enhanced potency of immune checkpoint inhibitors against poorly immunological solid tumors by immune stimulatory oncolytic adenoviruses-mediated remodeling of the tumor microenvironment

**DOI:** 10.1186/s10020-025-01223-4

**Published:** 2025-05-07

**Authors:** Hyo Min Ahn, Bo-Kyeong Jung, JinWoo Hong, Dayoung Hong, A-Rum Yoon, Chae-Ok Yun

**Affiliations:** 1 Department of Bioengineering, College of Engineering, Hanyang University, Seoul, Republic of Korea; 2 Institute of Nano Science and Technology (INST), Hanyang University, Seoul, Republic of Korea; 3 Hanyang Institute of Bioscience and Biotechnology (HY-IBB), Hanyang University, Seoul, Republic of Korea; 4 GeneMedicine Co., Ltd, 222 Wangsimni-Ro, Seongdong-Gu, Seoul, Republic of Korea

**Keywords:** Checkpoint inhibitors, Oncolytic adenovirus, Programmed cell death protein 1, Programmed death-ligand 1, Cytotoxic T lymphocyte-associated protein 4, Combination therapy

## Abstract

Immune checkpoint inhibitor (ICI) have shown promising results against a variety of solid tumors across clinical trials. However, ICI monotherapy is often ineffective in patients with non-immunogenic tumors that exhibit high level of immunosuppression and low level of tumor infiltrating lymphocytes. To address these limitations, we have investigated a combination of ICIs [anti-PD-1 antibody (αPD-1), anti-PD-L1 antibody (αPD-L1), or anti-CTLA-4 antibody (αCTLA-4)] with several different immune stimulatory oncolytic adenoviruses (Ads) expressing different combinations of antitumor cytokines or immune modulatory factors [e.g., (1) interleukin (IL)-12 and granulocyte-macrophage colony-stimulating factor (GM-CSF; RdB/IL12/GMCSF), (2) IL-12 and short hairpin ribonucleic acid (shRNA) targeting vascular endothelial growth factor (RdB/IL12/shVEGF), (3) IL-12 and decorin (RdB/IL12/DCN), (4) GM-CSF, and thymidine kinase (RdB/IL12/GMCSF-TK), or (5) IL-12, GM-CSF, and relaxin (RdB/IL12/GMCSF-RLX)] to overcome tumor-induced immunosuppression. Through comparative evaluation of combination therapy regimens, our findings have identified αPD-1 as the optimal ICI candidate to synergize with different oncolytic Ads to induce potent antitumor immune response against poorly immunological solid tumors.

**Supplementary Information:**

The online version contains supplementary material available at 10.1186/s10020-025-01223-4.

## Introduction

Cancer immunology has been an important topic for the basic and clinical research, since the discoveries of tumor antigens and adaptive immunity (Disis et al. 2009; 2001; Dougan and Dranoff 2009). Cancer immunotherapy harnesses the patient’s immune system, both innate and adaptive, to attack and abolish the tumors. Under normal biological conditions, the immune system is capable of recognizing and eradicating tumor cells. However, the tumors possess diverse molecular and cellular mechanisms to evade the host immune system (Meissner et al. 2005). Major cellular players in immune suppression are regulatory T cells (Tregs) and M2-polarized tumor-associated macrophages (Ugel et al. 2015). Downregulation of immune function by these immunosuppressive factors in cancer patients leads to poor disease management by immunotherapy (Kavanaugh and Carbone 1996). Consequently, the success of cancer immunotherapy requires amelioration of the immunosuppressive tumor microenvironment and restoration of antitumor immune function.

Currently, the induction of antitumor immunity in clinical setting is reliant on inhibitor-mediated blockade of immune checkpoints. Under normal physiological conditions, immune checkpoints are crucial for the maintenance of self-tolerance (that is, the prevention of autoimmunity) and also to protect tissues from damage when the immune system is responding to pathogenic infection (Pardoll 2012). However, the expression of immune checkpoint proteins can be dysregulated by tumors to evade immune recognition. The immune checkpoint-mediated regulation of effector T cell function is dependent on both co-stimulatory and co-inhibitory molecules. Most of the clinically approved immune checkpoint inhibitors (ICIs) are designed to obstruct the interaction between co-inhibitory receptors and their ligands; these drugs mainly target 3 molecules of immune checkpoint axis known as cytotoxic T lymphocyte-associated protein 4 (CTLA-4), programmed cell death protein 1 (PD-1), and programmed death-ligand 1 (PD-L1) that ultimately increase the activation of T cells.

These ICIs have demonstrated striking and long-lasting clinical responses in a subset of solid tumors, but only in a fraction of patients (Sharma and Allison 2015; Topalian et al. 2015). ICI as monotherapy is mostly ineffective in patients with ‘cold tumors’ that exhibit high levels of immunosuppression with a low level of tumor-infiltrating lymphocytes. Recent studies in patients reported that the intratumoral presence of cluster of differentiation (CD)8^+^ T cells and PD-1 and PD-L1 expression are positive predictive biomarkers for anti-PD-1 therapy (Tumeh et al. 2014). As PD-1 expression is generally localized on intratumoral T cells (Yao et al. 2013), it is not surprising that a preexisting T-cell response dictates anti-PD-1 treatment outcome. By blocking the ligation of PD-1 by its ligands, PD-1-expressing T cells were demonstrated to have enhanced cytotoxic, proliferation, and migration capacity (Yao et al. 2013).

In this regard, oncolytic virotherapy could be a promising candidate to synergize with ICIs in a combination therapy regimen, since several clinical trials and preclinical studies have successfully demonstrated that oncolytic viruses can mount a potent antitumor immune reaction against solid tumors by promoting tumor-specific T helper type 1 (Th1) response (Rojas et al. 2015; Ahn et al. 2016; Cerullo et al. 2010; Li et al. 2007). This antitumor immune boosting effects of oncolytic viruses are achieved in part by enhanced cross-presentation of tumor antigens, which are generated by the cytolytic activity of the virus, to immune cells and subsequent activation of antitumor T cells (Duraiswamy et al. 2013; Peggs et al. 2009; Simpson et al. 2013). Therefore, oncolytic viruses are attractive means to convert ‘cold tumors’ with highly immunosuppressive tumor microenvironments to ‘hot tumors’, ultimately making these tumors to be more responsive to other immunotherapeutics like ICIs. Among the oncolytic viruses, adenovirus (Ad) is a particularly promising vector for immunotherapy. Oncolytic Ad can express antitumor cytokines at high levels in tumor tissues due to its highly efficacious gene transfer efficiency and cancer-specific amplification of therapeutic genes, resulting from viral replication and secondary infection of neighboring tumor cells (Choi et al. 2012a). These attributes enable therapeutic cytokines to be expressed preferentially and in a sustained manner within tumor tissues, resulting in a prolonged antitumor effect and diminished toxicity compared with systemically administered recombinant cytokines (Choi et al. 2012a).

In this study, we examined the combined therapeutic efficacy of ICIs [anti-PD-1 antibody (αPD-1), anti-PD-L1 antibody (αPD-L1), or anti-CTLA-4 antibody (αCTLA-4)] and several different immune stimulatory oncolytic Ads expressing a different combination of antitumor cytokines or immune modulatory factors [Interleukin (IL)-12 and granulocyte-macrophage colony-stimulating factor (GM-CSF)-co-expressing Ad (RdB/IL12/GMCSF), IL-12 and short hairpin vascular endothelial growth factor (shVEGF)-co-expressing Ad (RdB/IL12/shVEGF), IL-12- and decorin (DCN)-co-expressing Ad (RdB/IL12/DCN), IL-12-, GM-CSF-, and herpes simplex virus thymidine kinase (TK)-co-expressing Ad (RdB/IL12/GMCSF-TK), or IL-12-, GM-CSF-, and relaxin (RLX)-co-expressing Ad (RdB/IL12/GMCSF-RLX)] to overcome the limitations of ICI monotherapy.

The therapeutic transgenes expressed by these oncolytic Ads were selected based on their ability to modulate the tumor microenvironment and enhance antitumor immune responses. For instance, IL-12 promotes cytotoxic T lymphocyte (CTL) and natural killer (NK) cell activation via interferon (IFN)-γ production, while GM-CSF facilitates the recruitment and activation of antigen-presenting cells (APCs) to induce tumor-specific adaptive immunity (Cheng et al. 2022; Kumar et al. 2022). In support, the combined expression of these two cytokines by oncolytic Ad was shown to induce robust antitumor immune response and adaptive immune memory formation against the tumor (Choi et al. 2012a), supporting their selection as key immunostimulatory factors in the present study. DCN as a therapeutic gene promotes the degradation of extracellular matrix (ECM) and attenuates transforming growth factor (TGF)-β-mediated immunosuppression to elevate CD4^+^ and CD8 T^+^ cells and simultaneously attenuate Treg accumulation in tumor tissues (Choi et al. 2010; Oh et al. 2017). TK is a well-characterized suicide gene, which can also function as a safety switch in diverse gene therapy applications (Morita et al. 2024; Zhao et al. 2021). For oncolytic virotherapy, TK as a therapeutic gene can enable selective ablation of virus-infected cells via ganciclovir administration when there is a serious adverse reaction after virus administration (Morita et al. 2024). Similar to DCN, RLX expression also promoted ECM degradation in the tumor microenvironment to improve therapeutic spread, immune cell accessibility, or macrophage polarization toward antitumor immune response (Yoon et al. 2024; Chang et al. 2021 Kim et al. 2006; Zhou et al. 2021). Further, the co-expression of RLX with antitumor cytokines (e.g., IL-12) by oncolytic adenovirus was shown to convert poorly immunogenic ‘cold’ tumors into ‘hot’ tumors, ultimately resensitizing these tumors to ICI therapy and exerting synergistic antitumor immune response when utilized in combination (Jung et al. 2020).

Our present findings demonstrate that immune stimulatory oncolytic Ad can prime these ‘cold tumors’ to be more responsive toward ICIs in 2 different immunocompetent animal models [murine (C57BL/6) and Syrian hamster] by actively promoting recruitment and infiltration of immune effector cells, ultimately conferring long-lasting antitumor immunity through the establishment of immunological memory function against the tumor. Through examination of multiple combination therapy regimens, we demonstrate that oncolytic Ad-mediated remodeling of both physical and immunological aspects of tumor microenvironment can be a promising strategy to enhance the potency of ICI against poorly immunological solid tumors.

## Results

### Therapeutic efficacy of oncolytic Ads in combination with αPD-L1 in poorly immunogenic solid tumor

**Fig. 1:**
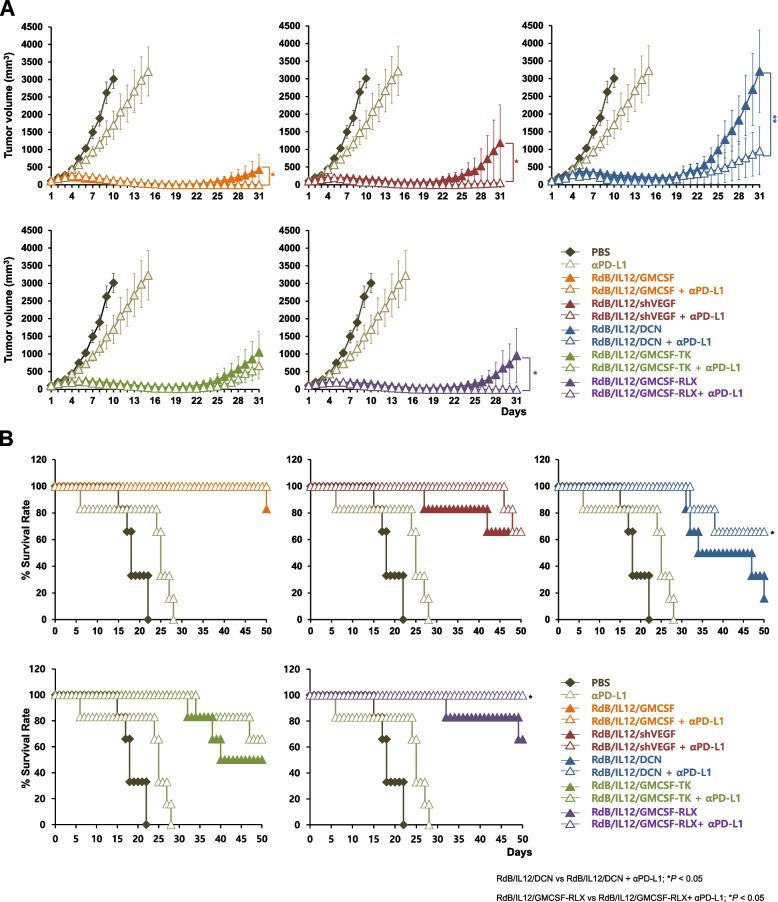
Antitumor effect and survival rate of oncolytic adenoviruses (Ads) combined with αPD-L1 in tumor-bearing mice. **A** The antitumor efficacy of oncolytic Ads combined with αPD-L1 was evaluated in B16-F10 tumor-bearing C57BL/6 mice over 31 days (initial tumor volume: 100 mm^3^). Mice received intratumoral injections of oncolytic Ads (5 × 10⁷ plaque-forming units; PFU) on days 1, 3, and 5. Concurrently, αPD-L1 (200 μg) was administered intraperitoneally on days 3, 6, and 9. Tumor volume was monitored and recorded every day until the end of the study (day 31). Significant tumor growth inhibition was observed in the combination groups. Data are presented as mean ± standard error (SE) (*n* = 6). *P < 0.05, **P < 0.01. **B** The survival rate of B16-F10 tumor-bearing mice treated with oncolytic Ads and αPD-L1 was assessed. Data are presented as mean ± SE (*n* = 6). *P < 0.05

To evaluate the therapeutic efficacy of oncolytic Ads in combination with αPD-L1, B16-F10 melanoma, which is a poorly immunogenic tumor (Xu et al. 2004), established in C57BL/6 mice were injected with oncolytic Ads (RdB/IL12/GMCSF, RdB/IL12/shVEGF, RdB/IL12/DCN, RdB/IL12/GMCSF-TK, or RdB/IL12/GMCSF-RLX) or one of these oncolytic Ads in combination with αPD-L1, along with phosphate-buffered saline (PBS) and αPD-L1 as controls. A backbone oncolytic Ad lacking therapeutic transgene, RdB, was omitted from the present experiment  since this virus only exerted negligible antitumor immune response compared to RdB/IL12/GMCSF, RdB/IL12/shVEGF, RdB/IL12/DCN, or RdB/IL12/GMCSF-RLX (Ahn et al. 2016; Oh et al. 2017; Jung et al. 2020). Oncolytic Ads (5 × 10^7^ plaque-forming units; PFU) were intratumorally administered on days 1, 3, and 5, whereas the αPD-L1 was intraperitoneally administered on days 3, 6, and 9. As shown in Fig. 1A, all mice in PBS and αPD-L1 control groups exhibited aggressive tumor growth, resulting in large tumors with average volume exceeding 3,000 mm^3^ by 10 and 15 days, respectively, following initial treatment. In contrast, significant tumor growth inhibition was observed in mice treated with the combination of αPD-L1 and RdB/IL12/GMCSF, RdB/IL12/shVEGF, RdB/IL12/DCN, RdB/IL12/GMCSF-TK, or RdB/IL12/GMCSF-RLX, showing 100.0%, 95.1%, 70.1%, 36.3% and 99.5% reduction in tumor burden compared to the respective oncolytic adenovirus at 31 days after initial treatment (*P < 0.5, **P < 0.01), respectively. The combination of αPD-L1 and RdB/IL12/GMCSF, RdB/IL12/shVEGF, RdB/IL12/DCN, RdB/IL12/GMCSF-TK, or RdB/IL12/GMCSF-RLX also resulted in 66.7%, 83.3%, 50.0%, 50.0%, and 83.3% tumor regression on day 50 post initial treatment, respectively (Table 1). In marked contrast, all mice in the PBS and αPD-L1 treatment groups neither achieved tumor regression nor survived on day 50. Moreover, a significant improvement in the survival rate of the mice treated with RdB/IL12/DCN + αPD-L1 or RdB/IL12/GMCSF-RLX + αPD-L1 was observed compared to their respective monotherapy groups (Fig. 1B; *P < 0.5). Among the evaluated oncolytic Ads and their combination therapy, the complete tumor regression rate was the highest for RdB/IL12/shVEGF or RdB/IL12/GMCSF-RLX combined with αPD-L1 at 83.3% (Table 1). Of the two viruses, RdB/IL12/GMCSF-RLX monotherapy exerted marginally more potent antitumor efficacy in comparison to RdB/IL12/shVEGF (Fig. 1A; mean tumor volume on day 31 was 955 versus 1185 mm^3^, respectively), and the long-term survival rate was also slightly improved when RdB/IL12/GMCSF-RLX was combined with αPD-L1 rather than RdB/IL12/shVEGF (Fig. 1B; 100% versus 83.3% survival rate at end-point). Based on these findings, RdB/IL12/GMCSF-RLX and its combination with ICIs were investigated at greater depth in subsequent experiments conducted in murine tumor models. Taken together, these results suggest that αPD-L1 in combination with immune stimulatory oncolytic Ads can prolong the survival of mice bearing poorly immunogenic tumors by eliciting a potent antitumor effect.

**Table 1:** Death rate and complete tumor regression percentages at day 50

day 50	PBS	RdB/IL12/GMCSF	RdB/IL12/shVEGF	RdB/IL12/DCN	RdB/IL12 /GMCSF-TK	RdB/IL12 /GMCSF-RLX
(-) αPD-L1	**0/6 (0.0%)**	**4/6 (66.7%)**	**4/6 (66.7%)**	**0/6 (0.0%)**	**2/6 (33.3%)**	**3/6 (50.0%)**
(+) αPD-L1	**0/6 (0.0%)**	**4/6 (66.7%)**	**5/6 (83.3%)**	**3/6 (50.0%)**	**3/6 (50.0%)**	**5/6 (83.3%)**
Death rate (%)						
day 50	**PBS**	**RdB/IL12/GMCSF**	**RdB/IL12/shVEGF**	**RdB/IL12/DCN**	**RdB/IL12** **/GMCSF-TK**	**RdB/IL12** **/GMCSF-RLX**
(-) αPD-L1	**6/6 (100%)**	**1/6 (16.7%)**	**2/6 (33.3%)**	**5/6 (83.3%)**	**4/6 (66.7%)**	**2/6 (33.3%)**
(+) αPD-L1	**6/6 (100%)**	**0/6 (0.0%)**	**1/6 (16.7%)**	**2/6 (33.3%)**	**3/6 (50.0%)**	**0/6 (0.0%)**

Complete disappearance of palpable or visible tumor was defined as “complete tumor regression”

B16-F10 tumor-bearing mice were treated with various oncolytic Ads, including RdB/IL12/GMCSF-RLX, RdB/IL12/GMCSF-TK, RdB/IL12/DCN, RdB/IL12/shVEGF, and RdB/IL12/GMCSF, with or without the addition of αPD-L1. The table summarizes the complete tumor regression and death rates on day 50. Data are presented as the number of mice with observed outcomes over the total number of treated mice, with corresponding percentages in parentheses

### Comparison of different ICIs in combination with RdB/IL12/GMCSF-RLX in poorly immunogenic solid tumor

**Fig. 2:**
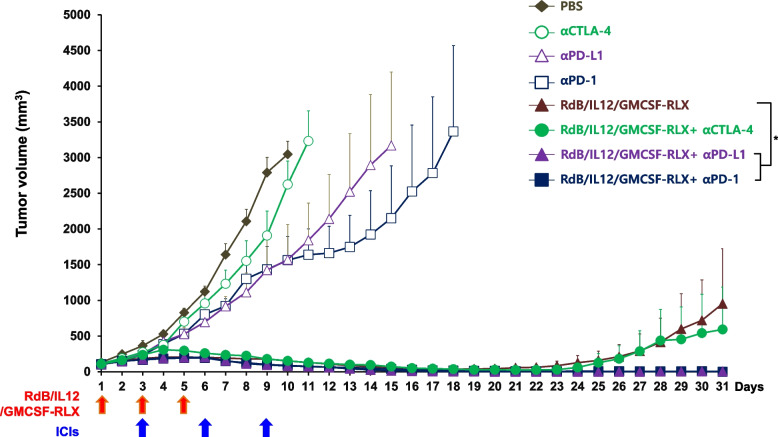
Therapeutic efficacy of RdB/IL12/GMCSF-RLX in combination with ICIs in poorly immunogenic solid tumor. The antitumor effect of each immune checkpoint inhibitor (ICI; αPD-1, αPD-L1, or αCTLA-4; 200 μg on days 3, 6, and 9) combined with RdB/IL12/GMCSF-RLX (5 × 10^7^ PFU administered on days 1, 3, and 5) in B16-F10 tumor-bearing C57BL/6 mice (mean initial tumor volume on day 1 was 100 mm^3^). Tumor volume was monitored and recorded every day until the end of the study (day 31). Arrows indicate the timing of the treatment administration. Data are represented the mean ± SE (*n* = 6). *P < 0.05

To assess which of the ICIs (αPD-1, αPD-L1, and αCTLA-4) synergizes better with an oncolytic Ad, B16-F10 melanoma tumors established in C57BL/6 mice were treated with RdB/IL12/GMCSF-RLX or RdB/IL12/GMCSF-RLX in combination with αPD-1, αPD-L1, or αCTLA-4, along with PBS or ICI monotherapy as controls. All mice in the PBS, αPD-1, αPD-L1, or αCTLA-4 control group showed aggressive tumor growth, resulting in large tumors with average volume exceeding 3,000 mm^3^ by 10, 18, 15, or 11 days post initial treatment, respectively (Fig. 2). These results indicate that ICIs alone have limited efficacy in this poorly immunogenic tumor model. In contrast, mice treated with RdB/IL12/GMCSF-RLX plus αPD-L1, αPD-1, or αCTLA-4 showed significantly inhibited tumor growth. There was 99.5%, 100.0%, and 37.9% reduction in tumor growth compared to the RdB/IL12/GMCSF-RLX monotherapy at 31 days after initial treatment, respectively. Of note, the combination of RdB/IL12/GMCSF-RLX with any of the ICIs (αPD-1, αPD-L1, or αCTLA-4) led to a higher complete tumor regression rate (100.0%, 83.3%, and 66.7%, respectively) in comparison to those treated with RdB/IL12/GMCSF-RLX monotherapy (50%, Table 2). Among the evaluated ICIs, αPD-1 in combination with oncolytic Ad co-expressing IL12, GMCSF and RLX induced highest number of complete tumor regression and most effective prevention of tumor recurrence, thus oncolytic Ad and αPD-1 combination therapy was chosen for more in-depth investigation in the subsequent experiments.

**Table 2:** Complete tumor regression and recurrence rates

	**PBS**	**αCTLA-4**	**αPD-L1**	**αPD-1**	**RdB/IL12/GMCSF-RLX**	**RdB/IL12/GMCSF-RLX** ** + αCTLA-4**	**RdB/IL12/GMCSF-RLX** ** + αPD-L1**	**RdB/IL12/GMCSF-RLX** ** + αPD-1**
Complete tumor regression (%)	**0/6** **(0.0%)**	**0/6** **(0.0%)**	**0/6** **(0.0%)**	**0/6** **(0.0%)**	**3/6** **(50.0%)**	**4/6** **(66.7%)**	**5/6** **(83.3%)**	**6/6** **(100.0%)**
Tumor recurrence (%)	**-**	**-**	**-**	**-**	**2/3** **(66.7%)**	**3/4** **(75.0%)**	**3/5** **(60.0%)**	**1/6** **(16.7%)**

^*^Recurrent tumors were first observed between day 5 and day 12 after the cancer cell rechallenge

B16-F10 tumor-bearing mice were treated with RdB/IL12/GMCSF-RLX monotherapy or in combination with ICIs, including αPD-1, αPD-L1, or αCTLA-4. The table displays the complete tumor regression and tumor recurrence rates. Data are presented as the number of mice with observed outcomes over the total number of treated mice, with corresponding percentages in parentheses

### Increased expression of IL-12, GM-CSF, RLX, and interferon (IFN)-γ in tumor tissues following RdB/IL12/GMCSF-RLX in combination with αPD-1 treatment

**Fig. 3:**
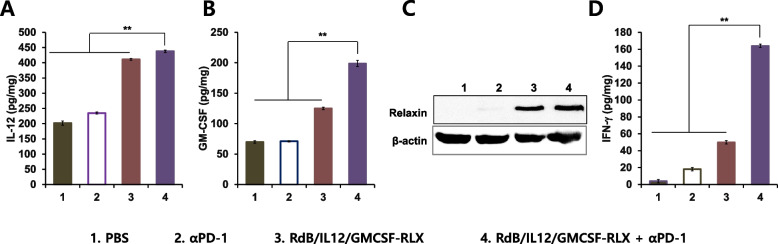
Local tumor expression of interleukin (IL)−12, granulocyte–macrophage colony-stimulating factor (GM-CSF), relaxin (RLX), and interferon (IFN)-γ. B16-F10 tumor-bearing mice were treated with PBS, αPD-1 (200 μg on days 3, 6, and 9), RdB/IL12/GMCSF-RLX (5 × 10⁷ PFU on days 1, 3, and 5), or the combination of αPD-1 and RdB/IL12/GMCSF-RLX. The tumor tissues were collected on day 12. Enzyme-linked immunosorbent assay (ELISA) or western blotting was used to quantify the levels of (**A**) IL-12, (**B**) GM-CSF, (**C**) RLX, and (**D**) IFN-γ in tumor tissues. Each data point represents the mean ± SE of IL-12, GM-CSF, and IFN-γ levels in the tumor tissues (*n* = 3); **P < 0.01

To examine the intratumoral expression levels of cytokines and RLX induced by different treatments, B16-F10 tumor tissues were harvested at 12 days after initial viral injection. As seen in Fig. 3A, tumors treated with RdB/IL12/GMCSF-RLX + αPD-1 showed highest concentrations of IL-12 (438 ± 4 pg/mg; **P < 0.01) with other groups showing lower IL-12 level in tumors; PBS (202 ± 7 pg/mg), αPD-1 (235 ± 3 pg/mg), or RdB/IL12/GMCSF-RLX (411 ± 3 pg/mg). Similarly, the tumors treated with combination of RdB/IL12/GMCSF-RLX + αPD-1 exhibited significantly higher levels of GM-CSF (199 ± 5 pg/mg) than other treatment groups (Fig. 3B; **P < 0.01); PBS (70 ± 2 pg/mg), αPD-1 (71 ± 1 pg/mg), or RdB/IL12/GMCSF-RLX (125 ± 2 pg/mg). RLX expression was detected in tumors treated with either RdB/IL12/GMCSF-RLX monotherapy or combination of RdB/IL12/GMCSF-RLX + αPD-1 (Fig. 3C), implying that all therapeutic genes can be expressed effectively by oncolytic Ad in tumor tissues.

IFN-γ, a key cytokine that prevents tumor progression and mediating tumor-specific immune responses (Dominiecki et al. 2005), was also evaluated in the tumor tissues. Figure 3D shows that the tumors treated with RdB/IL12/GMCSF + αPD-1 exhibited significantly higher level of IFN-γ (164 ± 2 pg/mg; **P < 0.01) than other treatment groups, resulting in 3.3- or 9.1-fold higher IFN-γ expression than RdB/IL12/GMCSF-RLX- or αPD-1-treated tumors (50 ± 2 pg/mg or 18 ± 2 pg/mg), respectively. Together, these findings suggest that the combination of immune-stimulatory oncolytic Ad and ICI can synergistically enhance the expression of antitumor cytokines in tumor microenvironment, leading to a robust antitumor immune response.

### Induction of a tumor-specific immune response through an increase in Th1/Th2 cytokine ratio and prevention of thymic atrophy

**Fig. 4:**
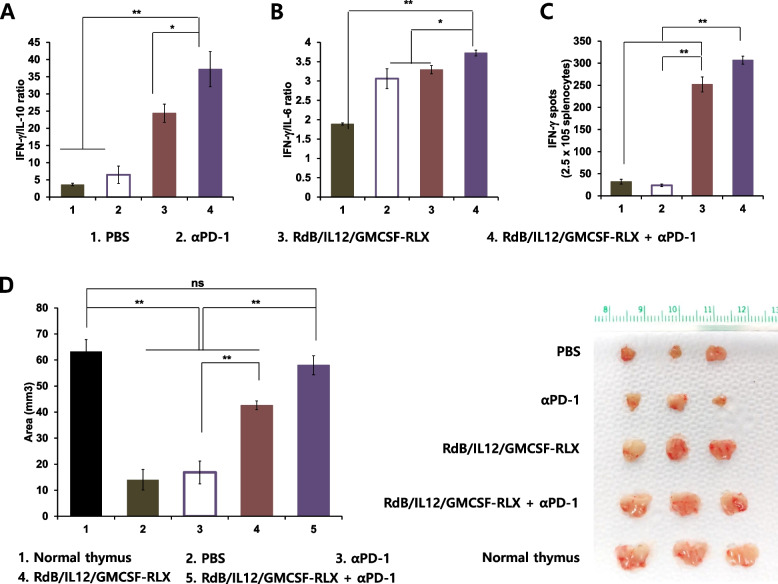
Assessment of IFN-γ/IL-6 and IFN-γ/IL-6 cytokine ratio and prevention of thymic atrophy. Splenocytes were harvested from B16-F10 tumor-bearing mice treated with PBS, αPD-1 (200 μg on day 3, 6, and 9), RdB/IL12/GMCSF-RLX (5 × 10^7^ PFU on days 1, 3, and 5), or αPD-1 plus RdB/IL12/GMCSF-RLX on day 12, and cocultured with irradiated B16-F10 tumor cells for 3 days in the presence of recombinant mouse IL-2. The Th1/Th2 CBA assay was performed to determine the ratio of (**A**) IFN-γ/IL-10 and (**B**) IFN-γ/IL-6 cytokine ratio in co-cultured supernatant. Each data point indicates the mean ± SE of three independent experiments, each performed with triplicate samples; *P < 0.05, **P < 0.01. **C** Splenocytes were collected from PBS, αPD-1, RdB/IL12/GMCSF-RLX, or αPD-1 plus RdB/IL12/GMCSF-RLX-treated mice at 12 days following the initial viral treatment, and then co-incubated with pre-irradiated B16-F10 cells for 18 h. The IFN-γ ELISpot assay was performed, and the spots were quantified using a computer-based immunospot system. Each value represents the mean spot number ± SE of triplicate experiments; **P < 0.01. **D** The image and area measurements of thymuses in mice treated with PBS, αPD-1, RdB/IL12/GMCSF-RLX, or the combination of αPD-1 and RdB/IL12/GMCSF-RLX; **P < 0.01

The shift from Th1 to Th2 cytokine expression has been shown to promote progression of malignant tumors (Gadducci et al. 2004; Smyth et al. 2004). Furthermore, a strong correlation between the potency of cancer therapeutics and Th1/Th2 cytokine ratio has been reported (Gadducci et al. 2004; Smyth et al. 2004; Katsikis et al. 1995). Therefore, we investigated whether RdB/IL12/GMCSF-RLX + ICI, which produced markedly higher amounts of most representative Th1 type cytokine (IL-12 and IFN-γ) than any other treatment group, could shift the tumor microenvironment from Th2 to Th1 immunity. Splenocytes of mice were harvested at 12 days post initial injection of oncolytic Ad and co-cultured with irradiated B16-F10 tumor cells for 3 days in the presence of recombinant mouse IL-2. The Th1/Th2 cytokine ratios (IFN-γ/IL-10 and IFN-γ/IL-6) were then analyzed in the co-cultured supernatant. As shown in Figs. 4A and 4B, the splenocytes from mice treated RdB/IL12/GMCSF-RLX + αPD-1 exhibited the highest IFN-γ/IL-10 and IFN-γ/IL-6 ratio among all treatment groups (*P < 0.05, **P < 0.01).

To further assess induction of tumor-specific immune response, both IFN-γ ELISpot assay and flow cytometric analysis were performed using splenocytes harvested from each treatment group. As shown in Fig. 4C, the frequency of IFN-γ-secreting immune cells appeared to be higher in mice treated with RdB/IL12/GMCSF-RLX compared to those treated with αPD-1 alone, suggesting that oncolytic Ad may play a more dominant role in promoting Th1-type immune activation. Notably, the co-treatment group (RdB/IL12/GMCSF-RLX + αPD-1) demonstrated the highest number of IFN-γ-secreting lymphocytes among all groups.

To further delineate which immune cell subsets induced the immune response, we performed flow cytometric analysis of splenocytes from B16-F10 tumor-bearing mice. In mice that received the combination therapy (RdB/IL12/GMCSF-RLX + αPD-1), the highest levels of CD3⁺CD4⁺IFN-γ⁺ and CD3⁺CD8⁺IFN-γ⁺ cell population were observed (Supplementary Figure). In support, CD3⁺CD4⁺CD69⁺ and CD3⁺CD8⁺CD69⁺ T cell populations were also observed at the highest levels in combination therapy group, suggesting that the treatment induced robust activation of T cells. In contrast, NK and Treg cell population levels remained similar across different treatment groups. Taken together, these results suggest that the antitumor effect of the combination therapy was primarily mediated by activation of T cells and an increase in tumor-specific Th1 immune responses.

Previous studies reported that thymic atrophy observed in tumor-bearing mice contributed to suppression of host immunity against the tumor (Carrio and Lopez 2009; Fu et al. 1989). These findings suggested that prevention of thymic atrophy could alleviate tumor-induced immunosuppression. Therefore, we first examined the effects of PBS, αPD-1, RdB/IL12/GMCSF-RLX, and RdB/IL12/GMCSF-RLX + αPD-1 on the mitigation of thymic atrophy in B16-F10 tumor-bearing mice. As shown in Fig. 4D, we observed thymic atrophy in mice treated with PBS, αPD-1, or RdB/IL12/GMCSF-RLX: the thymuses of these mice being significantly smaller than those of healthy mice (**P < 0.01). Notably, mice treated with RdB/IL12/GMCSF-RLX showed a significantly larger thymic area than those treated with αPD-1, indicating that oncolytic Ad can help mitigate thymic atrophy (**P < 0.01). Furthermore, the combination treatment of RdB/IL12/GMCSF-RLX + αPD-1 resulted in thymic sizes comparable to those of healthy and tumor-free mice, demonstrating that the combinatorial therapy effectively prevents thymic atrophy.

### Memory antitumor immune response following oncolytic Ads and ICIs combination therapy

**Fig. 5:**
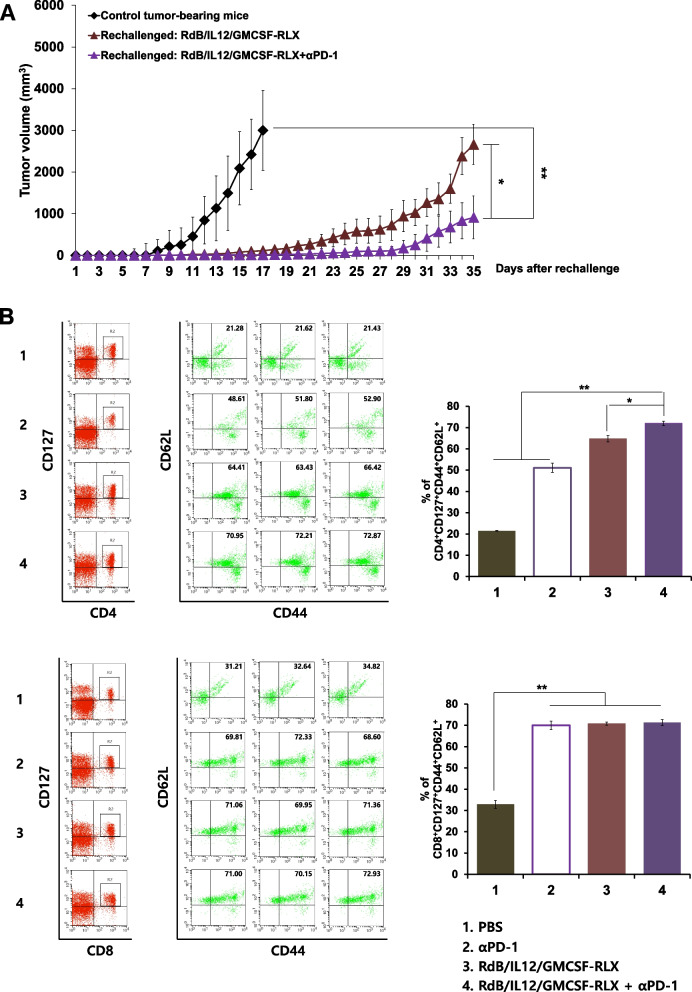
Tumor growth of rechallenged tumor and memory immune response. **A** B16-F10 tumor-bearing mice were treated with RdB/IL12/GMCSF-RLX alone or in combination with αPD-1 (RdB/IL12/GMCSF-RLX plus αPD-1). Mice with completely regressed primary tumors on day 50 were rechallenged by subcutaneously injecting 5 × 10^5^ B16-F10 cells into the left abdominal region. Control tumor-bearing mice were age-matched treatment naïve mice that were newly injected with B16-F10 cells to establish subcutaneous tumors. The growth of rechallenged tumors was monitored and recorded every day until the end of the study (day 35 after the rechallenge); *P < 0.05, **P < 0.01. **B** Percentage of CD44^high^CD62L^high^ T cells in CD4^+^CD127^+^ or CD8^+^CD127^+^ T cells was quantitated by flow cytometry. Splenocytes were harvested from B16-F10 rechallenged tumor-bearing mice on day 12 after treatment with PBS, αPD-1 (200 μg on day 3, 6, and 9), RdB/IL12/GMCSF-RLX (5 × 10^7^ PFU on days 1, 3, and 5), or αPD-1 plus RdB/IL12/GMCSF-RLX, and were analyzed after gating on the mononuclear lymphocyte population. CD4^+^CD127^+^ or CD8^+^CD127^+^ T cells were gated on and analyzed for the CD44^high^ and CD62L.^high^ cells subpopulations. Data points represent mean ± SE (*n* = 3). *P < 0.05, **P < 0.01

Immune memory function of memory B and T lymphocytes exposed to tumor antigens is essential for the induction of tumor-specific immune responses (Bachmann et al. 1997; Bachmann and Kopf 1999; 2009). To compare the antitumor immune memory function of mice treated with either oncolytic Ad monotherapy or combination therapy, mice that had achieved complete tumor regression from Fig. 1 were rechallenged with second round of tumor cell inoculation. As shown in Fig. 5A, all rechallenged tumors grew slower than the tumor of naive control mice without any previous treatment (**P < 0.01 vs. control tumor-bearing mouse). Importantly, the rechallenged tumor volume in the RdB/IL12/GMCSF-RLX + αPD-1-treated group was 65.7% smaller than those treated with RdB/IL12/GMCSF-RLX alone on day 35 after the rechallenge. Furthermore, tumor recurrence rates after rechallenging were significantly lower in mice treated with RdB/IL12/GMCSF-RLX + αPD-L1 compared to those treated with RdB/IL12/GMCSF alone. As shown in Table 3, the recurrence rate was 25.0% (1/4 mice) for the RdB/IL12/GMCSF + αPD-L1 group, whereas the RdB/IL12/GMCSF monotherapy group exhibited a 100% recurrence rate (4/4 mice). Similarly, mice treated with RdB/IL12/shVEGF in combination with αPD-L1 also showed 30% lower tumor recurrence rate compared to respective oncolytic Ad monotherapy that led to tumor recurrence in 50% of rechallenged mice (Table 3). These findings indicated that combining oncolytic Ads with αPD-L1 significantly reduced tumor recurrence.

**Table 3:** Complete tumor regression and recurrence rates in tumor-bearing mice treated with oncolytic Ad combined with ICIs

Group	Complete tumor regression rate (primary tumor)	Tumor recurrence rate (rechallenged tumor)
RdB/IL12/GMCSF	**4/6 (66.7%)**	**4/4 (100.0%)**
RdB/IL12/GMCSF + αPD-L1	**4/6 (66.7%)**	**1/4 (25.0%)**
RdB/IL12/shVEGF	**4/6 (66.7%)**	**2/4 (50.0%)**
RdB/IL12/shVEGF + αPD-L1	**5/6 (83.3%)**	**1/5 (20.0%)**

The table shows the complete tumor regression rates of the primary B16-F10 tumor after treatment with either RdB/IL12/GCMSF or RdB/IL12/shVEGF monotherapy or in combination with αPD-L1. For the analysis of tumor recurrence rate (rechallenged tumors), mice with complete tumor regression on day 50 were subsequently rechallenged with B16-F10 cells via subcutaneous administration. Any mice that showed establishment and growth of rechallenged tumors up to 35 days after tumor rechallenge were considered to be positive for tumor recurrence. Data are presented as the number of mice with observed outcomes over the total number of treated mice, with corresponding percentages in parentheses

We further evaluated the memory immune response by characterizing the phenotype of splenocytes from mice treated with PBS, αPD-1, RdB/IL12/GMCSF-RLX, or RdB/IL12/GMCSF-RLX + αPD-1. Splenocytes were harvested 12 days post initial injection of Ad and were used to assess the population of central memory T cell based on CD44, CD62L and CD127 expression (central memory T cell: CD44^high^CD62L^high^CD127^high^) (Martín-Fontecha et al. 2008; Stemberger et al. 2007). As shown in Fig. 5B, the populations of CD4^+^ central memory T cells were significantly increased in the mice treated with RdB/IL12/GMCSF-RLX + αPD-1 compared with the other group (PBS, αPD-1, and RdB/IL12/GMCSF-RLX) (*P < 0.05, **P < 0.01). In addition, the populations of CD8^+^ central memory T cells were significantly increased in all treated groups compared with PBS group (**P < 0.01). However, there was no difference among other treatment groups. Together, these results demonstrate that RdB/IL12/GMCSF-RLX effectively promotes immune memory formation, and that this memory is significantly augmented when RdB/IL12/GMCSF-RLX is combined with αPD-1.

### Optimizing antitumor efficacy of combination therapy against recurrent tumors

Tumor recurrence remains a major etiology of the morbidity and mortality in cancer patients (Mitra et al. 2015; Wal et al. 2016; Baker et al. 2005). In the case of melanoma, patients successfully treated for localized melanoma are at 5- to tenfold higher risk of melanoma recurrence than people without prior history of melanoma (Welch et al. 2005; Youlden et al. 2014). Moreover, recurrent tumors are highly resistant to anti-cancer drugs and are capable of evading immune surveillance (Mitra et al. 2015).

**Fig. 6:**
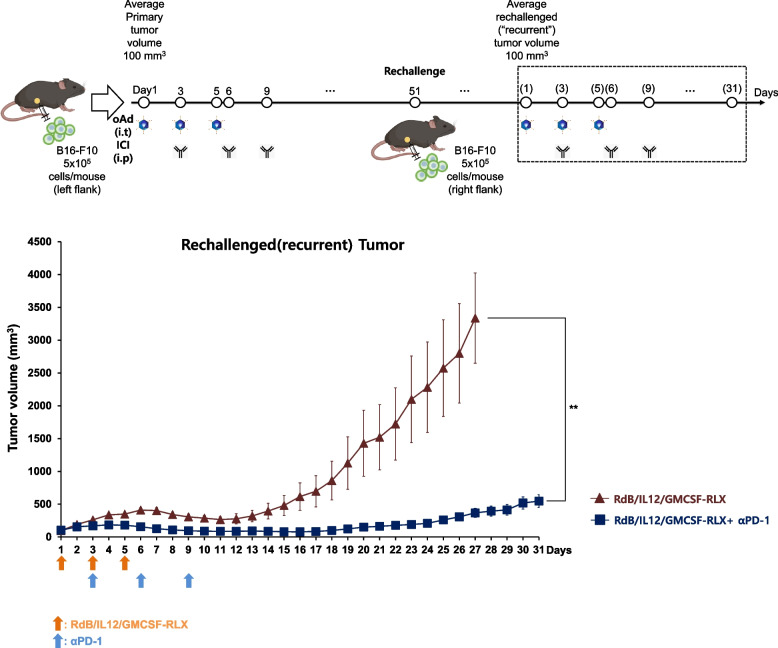
Antitumor efficacy in recurrent tumors. C57BL/6 mice that had achieved complete tumor regression after the initial treatment with RdB/IL12/GMCSF-RLX or RdB/IL12/GMCSF-RLX + αPD-1 were rechallenged with 5 × 10^5^ B16-F10 cells subcutaneously on the right flank on day 50 after the first virus administration. When the rechallenged tumors reached an average initial volume of approximately 100 mm.^3^ (designated as day 1 of the second treatment cycle), mice were treated with RdB/IL12/GMCSF-RLX (1 × 10⁷ PFU/intratumoral injection, fivefold lower than the initial treatment) on days 1, 3, and 5 as monotherapy or in combination with αPD-1 (200 μg/intraperitoneal injection) on days 3, 6, and 9. Secondary (“recurrent”) tumor volume was measured daily for 31 days following the start of the second treatment cycle. Data points represent mean ± SE (*n* = 5). **P < 0.01

To evaluate whether repeat administration of RdB/IL12/GMCSF-RLX in combination with αPD-1 can be an effective treatment against recurrent tumors, a tumor model emulating tumor recurrence was established by tumor rechallenge protocol. Briefly, the primary B16-F10 tumors were established on the left flank of mice, and these mice either were treated with RdB/IL12/GMCSF-RLX monotherapy (5 × 10⁷PFU/dose) or in combination with αPD-1 as described in Fig. 2, and mice that remained in complete tumor regression at day 51 were rechallenged with B16-F10 cells on the opposite flank to establish the recurrent tumors. When the recurrent tumors reached 100 mm^3^ (designated as day 1 of the second treatment cycle), mice received a second round of treatment with RdB/IL12/GMCSF-RLX (1 × 10⁷ PFU/intratumoral injection on days 1, 3, and 5) and αPD-1 (200 μg/intraperitoneal injection on days 3, 6, and 9). Notably, the viral dose used in this second cycle was fivefold lower than that used in the initial treatment, aiming to assess whether initial treatment-induced alteration of the tumor microenvironment can lead to more robust induction of secondary antitumor immune response. As shown in Fig. 6, secondary treatment of recurrent tumor-bearing mice with RdB/IL12/GMCSF-RLX + αPD-1 led to more robust reduction in tumor burden compared to the RdB/IL12/GMCSF-RLX monotherapy at 31 days after the initial treatment (**P < 0.01). These results suggested that the combination of ICI and oncolytic Ad can lead to potent tumor growth inhibition of both primary and recurrent tumors. Notably, complete remission was achieved in two of six mice treated with RdB/IL12/GMCSF-RLX + αPD-1 combination, while no mice in the RdB/IL12/GMCSF-RLX monotherapy group experienced complete regression. Taken together, these results suggest that oncolytic Ads combined with αPD-1 showed potent antitumor efficacy in relapsed cancer model, providing a promising therapeutic strategy for managing recurrent tumors.

### Therapeutic efficacy of the oncolytic Ad co-expressing IL-12 and DCN combined with ICIs in HaP-T1 tumor-bearing Syrian hamster model

Pancreatic cancer is known to be a highly aggressive, metastatic, poorly immunogenic, and desmoplastic, making it notoriously difficult to treat (Yadav and Lowenfels 2013; Vincent et al. 2011; Siegel et al. 2014). Excessive desmoplastic reactions in pancreatic tumors lead to the formation of a dense ECM, acting as a barrier against the diffusion of anticancer therapeutics and host immune cells (Neoptolemos et al. 2004; Haller 2002; Acerbi et al. 2015). To overcome these limitations, we have utilized immune stimulatory oncolytic Ad (RdB/IL12/DCN), which co-expresses IL-12 and DCN, to simultaneously degrade tumor ECM and reverse pancreatic tumor-induced immunosuppression by blocking TGF-β synthesis and activity (Oh et al. 2017; Heino et al. 1988; Kähäri et al. 1995; Mukhopadhyay et al. 2010). Additionally, we have utilized Syrian hamster pancreatic tumor model for the evaluation of RdB/IL12/DCN in combination with ICIs (αPD-1, αPD-L1, or αCTLA-4). Syrian hamster model was chosen due to its permissiveness toward human serotype 5 Ad replication, which enable more complete modeling of the complex interactions among the virus, tumor, and host immune system compared to murine tumor models that are not permissive to Ad replication (Thomas et al. 2006a). Despite several advantageous attributes of Syrian hamster model, it should be noted that there are limited selection of experimental reagents available. Due to this limitation, Syrian hamster-specific ICIs were not available for purchase at the time of this study and mouse-reactive ICIs were utilized during the experiment.

**Fig. 7:**
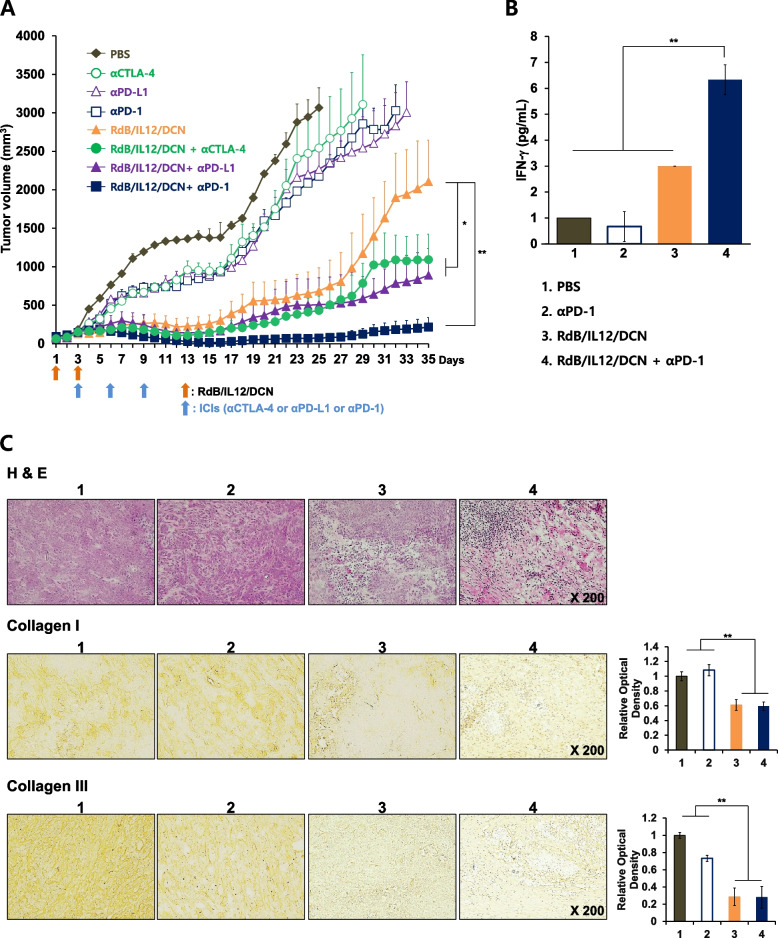
Antitumor efficacy of RdB/IL12/DCN with αPD-1 in HaP-T1 tumor-bearing Syrian hamster model. **A** Antitumor effect of RdB/IL12/DCN combined with αPD-1, αPD-L1, or αCTLA-4 in Hap-T1 tumor-bearing Syrian hamsters. Syrian hamsters were subcutaneously injected with HaP-T1 cells to establish pancreatic tumors. When the average tumor reached 100 mm^3^, the tumors were injected with 2 × 10^7^ PFU of RdB/IL12/DCN (day 1 and 3) or 700 μg of αPD-1 (day 3, 6, and 9). Tumor volume was measured every day until the end of the study (day 35). *P < 0.05 for RdB/IL12/DCN versus RdB/IL12/DCN + αCTLA-4 and RdB/IL12/DCN + αPD-L1. **P < 0.01 for RdB/IL12/DCN versus RdB/IL12/DCN + αPD-1. Data points represent mean ± SE (*n* = 4); **P < 0.01. **B** Hap-T1 tumor tissues were collected 12 days post-initial treatment with PBS, αPD-1 (700 μg on day 3, 6, and 9), RdB/IL12/DCN (2 × 10^7^ PFU on days 1 and 3), or RdB/IL12/DCN plus αPD-1. ELISA was performed to quantitate the levels of hamster IFN-γ in tumor tissues. Each data point represents the mean ± SE (*n* = 3); **P < 0.01. **C** Hematoxylin and eosin (H&E)-stained sections of HaP-T1 tumor tissues treated with PBS, αPD-1, RdB/IL12/DCN, or RdB/IL12/DCN plus αPD-1. Immunohistochemical staining of collagen I and III in HaP-T1 tumor tissues. Original magnification, × 200

Once the HaP-T1 tumors reached an average tumor volume of 60 to 100 mm^3^ (day 1), the hamsters were treated with RdB/IL12/DCN (2 × 10^7^ PFU on day 1 and 3) in combination with or without ICIs (αPD-1, αPD-L1, or αCTLA-4; 700 μg on day 3, 6, and 9), along with PBS or ICI alone as controls. As shown in Fig. 7A, PBS-treated tumors exhibited robust growth with average volume exceeding 3,000 mm^3^ by 25 days following initial treatment. All ICI monotherapy groups attenuated tumor growth in comparison to PBS control, but these effects was markedly inferior to those achieved using RdB/IL12/DCN monotherapy. Importantly, RdB/IL12/DCN in combination with ICIs (αPD-1, αPD-L1, or αCTLA-4) induced superior tumor growth inhibition than respective ICI or oncolytic Ad monotherapies, showing preliminary evidence that mouse-reactive αPD-1, αPD-L1, or αCTLA-4 may exert therapeutic effect in Syrian hamster tumor model. In support, RdB/IL12/GMCSF-RLX in combination with same mouse-reactive αPD-1 was shown to exert synergistic antitumor effect against Hap-T1 and HP-1 Syrian pancreatic tumor models via robust immune activation (Jung et al. 2020). Of note, oncolytic Ad in combination with αPD-1 exhibited more potent tumor growth inhibition than combination therapy groups using different ICIs (αPD-L1 or αCTLA-4), elucidating that PD-1 blockade is the optimal ICI candidate to be used in conjunction with an oncolytic Ad in pancreatic cancer.

To evaluate cancer-specific immune response, splenocytes from hamsters treated with PBS, αPD-1, RdB/IL12/DCN, or RdB/IL12/DCN in combination with αPD-1 were harvested on the 11 days after the first virus injection then co-cultured with irradiated HaP-T1 cancer cells. After 72 h, the supernatant was harvested and the level of IFN-γ secreted by cancer-specific lymphocytes was measured by enzyme-linked immunosorbent assay (ELISA). As shown in Fig. 7B, IFN-γ expression was not increased significantly in αPD-1-treated group compared to PBS-treated group, suggesting that ICI alone could not sufficiently induce antitumor immune response in a poorly immunogenic pancreatic cancer. In contrast, both RdB/IL12/DCN and its αPD-1combination therapy group resulted in evidently increased IFN-γ expression, which was 2.1-fold higher in RdB/IL12/DCN + αPD-1 group than RdB/IL12/DCN monotherapy group (**P < 0.01). Together, these results suggest that immune stimulatory cytokine-expressing oncolytic Ad can induce cancer-specific immune response in poorly immunogenic tumors and this effect could be enhanced by co-administration with ICI.

Histological analysis revealed markedly enhanced tumor necrosis in tumor tissues treated with RdB/IL12/DCN + αPD-1 in comparison with PBS-, αPD-1- or RdB/IL12/DCN-treated tumor tissues (Fig. 7C). In addition, the effects of DCN on the expression of major ECM components (collagen I and III) of tumor tissues were evaluated by immunohistochemistry. Image analysis of immunohistochemical staining revealed significantly reduced collagen I and III in RdB/IL12/DCN or RdB/IL12/DCN + αPD-1-treated tumor tissue compared to PBS- or αPD-1-treated tumor tissues. The reduced expression of collagen I and III were semi-quantitatively measured with ImageJ image analysis software (Fig. 7C; **P < 0.01). These data strongly suggest that expressions levels of major ECM components, such as collagen I and III, are significantly decreased by DCN overexpression in HaP-T1 tumor tissues, thereby enhancing the therapeutic potential of RdB/IL12/DCN in overcoming the desmoplastic barrier of pancreatic tumors.

### Tumor rechallenge in Syrian hamsters with complete tumor regression after treatment with oncolytic Ad co-expressing IL-12 and DCN and ICIs

**Fig. 8:**
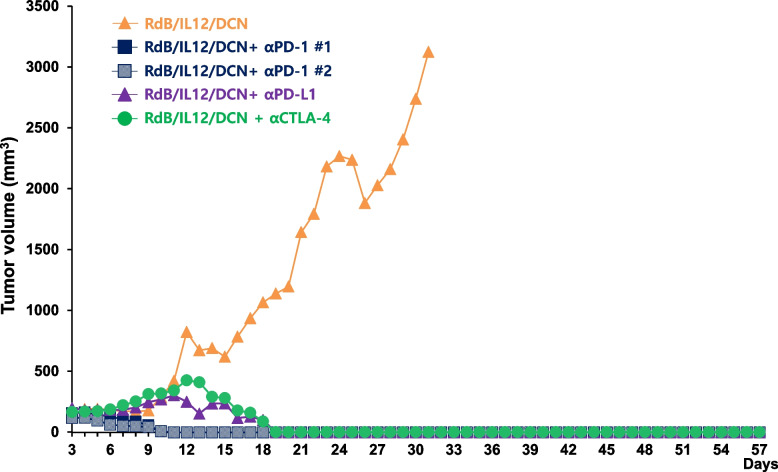
Rechallenged tumor growth in Syrian hamster model. Subcutaneous Hap-T1 tumor-bearing Syrian hamsters (initial mean tumor volume = 100 mm^3^) were treated with RdB/IL12/DCN alone or in combination with αPD-1, αPD-L1, or αCTLA-4 (from Fig. 7). Syrian hamsters with complete regression of the primary tumors on day 49 were rechallenged with 3 × 10.^6^ HaP-T1 cells into the left abdominal region by subcutaneous administration. Rechallenged tumor growth was monitored over time. The number of animals that was rechallenged from each treatment group: RdB/IL12/DCN (*n* = 1), RdB/IL12/DCN + αPD-1 (*n* = 2), RdB/IL12/DCN + αPD-L1 (*n* = 1), and RdB/IL12/DCN + αCTLA-4 (*n* = 1)

Systemic memory immune response is crucial for the inhibition or prevention of tumor metastasis and tumor relapse (Seth et al. 2014; Kim et al. 2015; Adema et al. 2017; Lee 1998). Thus, systemic adjuvant therapies like immunotherapy are necessary to target these residual tumor cells effectively. To assess the systemic antitumor immune response induced by RdB/IL12/DCN or RdB/IL12/DCN combined with ICIs, Syrian hamsters that had undergone tumor regression were rechallenged with HaP-T1 tumor cells in the left flank on day 50 after the initial treatment. The formation of rechallenged tumors was then observed on day 3 following tumor cell injections in treatment groups (Fig. 8). Notably, all rechallenged tumors in  combination treatment groups, including RdB/IL12/DCN + ICIs, were completely regressed, indicating a strong systemic antitumor response. In contrast, tumors in the RdB/IL12/DCN-only treated group showed significant growth, with the average tumor volume reaching 3,124 mm^3^ by day 31 post-rechallenge. Together, these results highlight that the combination of RdB/IL12/DCN with ICIs can confer long-lasting antitumor immune response through development of antitumor immunological memory.

## Discussion

It is now increasingly accepted that cancer cells closely interact with the ECM, stromal cells, and immune cells of an immunosuppressive tumor microenvironment in which tumor cells could adapt and grow with a lower likelihood of detection and eradication by host immunosurveillance (Hanahan and Coussens 2012). The immune escape mechanism within the neoplastic microenvironment is a crucial barrier limiting the success of immunotherapeutics (Dranoff 2004). In addition, the ECM, which acts as a physical barrier to anticancer drugs within the tumor, has also been identified as a major factor in preventing immunotherapeutic from dispersing throughout the tumor (Shields et al. 2012; Li et al. 2019). To address these challenges, we have comparatively evaluated different combinations of oncolytic Ads and ICIs.

Our findings showed that the therapeutic effect of oncolytic Ad co-expressing IL-12 and other therapeutic genes can elicit a potent antitumor effect against poorly immunogenic murine melanoma (Fig. 1–2), despite majority of murine tumors being poorly permissive to Ad replication (Thomas et al. 2006b; Ganly et al. 2000). These results suggested that the therapeutic effects were predominantly mediated by virus- and therapeutic gene-induced immune activation rather than direct viral oncolysis. Others have similarly noted that the replication-induced cytolytic effect may only contribute to a small portion of the therapeutic effect of oncolytic viruses (Davola and Mossman 2019; Baracuhy et al. 2024).

The therapeutic effect of oncolytic Ads was greatly improved by combination with ICIs (αPD-1, αCTLA-4, or αPD-L1) in both poorly immunogenic murine melanoma or Syrian hamster pancreatic tumor models that were generally unresponsive to ICI monotherapy (Fig. 1, 2, and 7). Among the tested ICIs, αPD-1 showed the strongest synergy with immune stimulatory oncolytic Ads, whereas combination with αCTLA-4 led to the lowest therapeutic benefit, in both tumor-bearing mice and Syrian hamsters (Table 2, Fig. 2 and 7). The superior synergistic effect of ICIs inhibiting the PD-1/PD-L1 signaling axis likely attributed to several factors. Briefly, it is well-established that cancer immunotherapeutics, like cytokines and oncolytic Ads, have been shown to elevate PD-1 expression in T cells or increase PD-1^+^ T cell population in the tumor tissues (Liu et al. 2024; 2020; Jiang et al. 2017; Cervera-Carrascon et al. 2020), and increased presence of Th1 cytokine IFN-γ led to PD-L1 upregulation by tumor cells (Tanoue et al. 2017; Boyerinas et al. 2015). These findings suggested that oncolytic Ad-mediated upregulation of PD-1/PD-L1 signaling axis in the tumor microenvironment may work synergistically with αPD-1 and αPD-L1. In comparison, the effect of oncolytic Ad and its interaction with CTLA-4 regulation or CTLA-4 inhibitor remain poorly understood, since only few studies have been reported to date (Yang et al. 2019; Zhang et al. 2022; Du et al. 2014). Alternatively, the primary site of T cell regulation by PD-1 has been reported to occur in a localized manner in the peripheral tissues, like the tumor, whereas CTLA-4 predominantly was shown to exert its function within the lymphoid organs (Buchbinder and Desai 2016; Goleva et al. 2021). As intratumorally administered oncolytic Ads primarily exert their therapeutic effect in the tumor tissues, the overlapping site of T cell activation by αPD-1 could be more beneficial than systemic immune activation by CTLA-4 in controlling primary tumors. Lastly, the strong synergy between oncolytic Ads and αPD-1 observed in the present study against poorly immunogenic melanoma and pancreatic cancer could also be clinically relevant, since PD-1 inhibitor, pembrolizumab, has shown superior efficacy over αCTLA-4, ipilimumab, in advanced melanoma patients and it is currently the only ICI approved for the treatment of pancreatic cancer by United States Food and Drug Administration (Long et al. 2024; Yoon et al. 2023).

The expression level of IL-12, GM-CSF, and RLX was significantly elevated in tumor tissues treated with RdB/IL12 or RdB/IL12/GMCSF-RLX + αPD-1 in tumor tissues (Figs. 3A to C). IL-12 is known to stimulate IFN-γ production from activated T and natural killer (NK) cells (Ahn et al. 1997). In agreement with previous findings, we observed elevated expression levels of the Th1 cytokine IFN-γ in mice treated with RdB/IL12/GMCSF-RLX in combination with αPD-1, both in tumor tissues and in splenocytes co-cultured with irradiated B16-F10 cells (Fig. 4C). To further explore which immune subsets may contribute to this response, we performed additional flow cytometry analysis (Supplementary Figure). While not statistically significant, the data suggested an upward trend in CD3⁺CD4⁺IFN-γ⁺ Th1 cells in the combination group, implying a possible role of Th1-type immunity in mediating the therapeutic effect. Similarly, CD3⁺CD8⁺CD69⁺ effector CTLs also showed a mild increase in the virus-treated groups, though without reaching statistical significance. Taken together, these results point toward a potential contribution of enhanced Th1-mediated antitumor immunity in the observed treatment efficacy, with a supportive role from CTLs.

In our results, the combination of ICI with immune stimulatory oncolytic Ad has led to elevation of Th1/Th2 cytokine ratio (IFN-γ/IL-10 and IFN-γ/IL-6) (Figs. 4A and B), implying that combination therapy can revert tumor-mediated immunosuppression by simultaneous boosting of Th1 cytokine expression and reduction in Th2 cytokines. Additionally, the combination therapy-treated mice possessed healthy thymuses, which showed no signs of visible atrophy, that were comparable to those of healthy mice without tumors (Fig. 4D), further supporting our claim that combined treatment can revert immunosuppressive conditions of tumors to an immunologically functional state. Indeed, our group and others have reported that thymic atrophy, which can suppress T cell development and T cell-mediated immune response, occurs in tumor-bearing mice (Ahn et al. 2016; Carrio and Lopez 2009; Fu et al. 1989; Lopez et al. 2002).

The formation of immune memory induced by GM-CSF was synergistically enhanced when combined with IL-12 (Choi et al. 2012b). In the present study, the growths of rechallenged tumors were suppressed in mice that had rejected the primary tumors following the treatment with oncolytic Ads (RdB/IL12/GMCSF, RdB/IL12/shVEGF, or RdB/IL12/GMCSF-RLX) combined with αPD-L1 compared with oncolytic Ad monotherapy (Fig. 5A and Table 3). Moreover, the splenocytes from mice treated with RdB/IL12/GMCSF-RLX + αPD-1 had a greater population of central memory CD4^+^ T cells (Fig. 5B). This strong immunological memory induced by treatment with RdB/IL12/GMCSF-RLX + αPD-1 may suppress local solid tumor growth and prevent recurrence.

This assertion is supported by previous works by our group and others demonstrating that degradation of tumor ECM positively correlates with enhanced accumulation of systemically administered cancer therapeutics (2020; Li et al. 2019; Yun 2008; Zhang et al. 2023; Kim et al. 2023; Ikeda-Imafuku et al. 2022). In our results, RdB/IL12/DCN, which was shown to attenuate major ECM components (Fig. 7C), induced potent antitumor effect when combined with αCTLA-4, αPD-L1, or αPD-1 (Fig. 7a). These findings are in line with previous reports that demonstrated that either DCN or RLX gene expression in tumor tissues led to effective degradation of ECM, which ultimately led to higher level of intratumoral immune cell infiltration or distribution of co-administered therapeutics (Oh et al. 2017; Jung et al. 2020; Li et al. 2019). These previous findings suggested that the RdB/IL12/GMCSF-RLX- or RdB/IL12/DCN-mediated expression of ECM degrading molecules in the present report may improve the intratumoral distribution of ICIs, while the expression of potent antitumor cytokine IL-12 inflamed poorly immunogenic tumor microenvironment to induce synergistic effect (Fig. 2–4, and 7).

As an alternative to a combination therapy regimen, recent studies have highlighted that oncolytic Ad-mediated expression of ICI as therapeutic gene is also a feasible option to combine oncolytic virotherapy and ICI into a single therapeutic modality (Rosewell Shaw et al. 2017; Xie et al. 2023). For instance, the combination of PD-L1-targeted minibody-expressing oncolytic Ad in combination with chimeric antigen receptor (CAR)-T cell therapy was shown to be more efficacious than the combination therapy utilizing a systemically administered PD-L1 IgG therapy (Tanoue et al. 2017), showcasing that oncolytic Ad-induced ICI expression can be superior to more conventional ICI treatment regimen in some biological settings. Despite several promising attributes, several potential limitations and unknowns must be investigated further before the clinical translation of this approach. For instance, the relatively large genetic size of antibodies (150 kDa corresponding to roughly 4,000 base pairs) can be problematic for most oncolytic Ad platforms due to packaging capacity limitation (Lu and Barry 2022), which may lead to attenuation in viral replication capacity and prevent insertion of other therapeutic genes. Additionally, it is extremely difficult to accurately predict how much ICI can be produced and remain in systemic circulation after oncolytic Ad injection due to the highly heterogenic nature of clinical tumors. In contrast, the pharmacodynamics of systemically administered ICIs are well-documented across multiple clinical trials (Centanni et al. 2019; Liu and Yu 2023), making it easier to predict and regulate ICI-mediated therapeutic effects when utilized in combination therapy approaches. As there are many unanswered questions, future studies that compare ICI-expressing oncolytic Ad versus oncolytic Ad and ICI combination therapy are eagerly awaited.

In conclusion, this study evaluates various ICI combinations with immune-stimulatory oncolytic Ads, using different transgene pairings in both Ad replication-permissive and poorly permissive immunocompetent tumor models. The combination of αPD-1 with RdB/IL12/GMCSF-RLX showed the most durable and potent antitumor immune response, suggesting that RdB/IL12/GMCSF-RLX can sensitize ICI-refractory cold tumors to checkpoint blockade. This multidimensional approach reprograms the TME into a more responsive state by remodeling both its physical and immunological aspects, providing a promising strategy to enhance ICI efficacy against poorly immunogenic solid tumors.

## Materials and methods

### Cell lines and cell culture

Dulbecco’s modified Eagle’s medium (PAN Biotech, Dorset, UK) supplemented with 10% fetal bovine serum (FBS; Alpha bioregen, Boston, MA), L-glutamine (2 mmol/L), penicillin (100 IU/mL), and streptomycin (50 mg/mL) was used as the culture medium. HEK293 (human embryonic kidney cell line expressing the Ad E1 region) and B16-F10 (murine melanoma cell line) were obtained from the American Type Culture Collection (Manassas, VA). HaP-T1 (hamster pancreatic cancer cell line) cells were kindly provided by Dr. Masato Abei (University of Tsukuba, Ibaraki, Japan). All cell lines tested negative for *Mycoplasma* when tested by Hoeschst dye staining (MP Biomedicals, Irvine, CA), cell culture, and polymerase chain reaction.

### Virus preparation

Construction and generation of RdB/IL12/GMCSF, RdB/IL12/shVEGF, and RdB/IL12/DCN were described previously (Ahn et al. 2016; Oh et al. 2017; Choi et al. 2012b). To generate an oncolytic Ad that expresses GM-CSF and TK at the E3 region of RdB/IL12 (Choi et al. 2012b), the murine GM-CSF-IRES complementary DNA (provided by Bank for Cytokine Research, Chonbuk National University) was generated using following primer pairs (sense: 5’-acggatccaccatgtggctgcagaatttacttttc-3’; antisense: (5’–caagatctttatcatcgtgtttttcaaaggaaaa–3’). The resulting polymerase chain reaction product was inserted into the pSP72 dEΔ3/CMV Ad E3 shuttle vector (Yun et al. 2005), generating pSP72 dE3/CMV/GMCSF-IRES, and TK gene from BamHI-digested pSP72 dE3/CMV-TK inserted into the pSP72 dE3/CMV/GMCSF-IRES, generating pSP72 dE3/CMV/GMCSF-IRES-TK Ad E3 shuttle vector. To construct an oncolytic Ad expressing IL-12 and GMCSF-IRES-TK at the E1 and E3 regions, respectively, the newly constructed pSP72 dE3/CMV/GMCSF-IRES-TK was co-transformed with the Ad total vector pRdB/IL12 (Choi et al. 2012b) at the E3 region into Escherichia coli BJ5183 for homologous recombination, generating the pRdB/IL12/GMCSF-TK Ad vector. To generate an oncolytic Ad expressing IL-12 and GMCSF-IRES-RLX at the E1 and E3 regions (pRdB/IL12/GMCSF-RLX), respectively, the newly constructed pSP72 dE3/CMV/GMCSF-IRES-RLX was co-transformed with the Ad total vector pRdB/IL12 (Choi et al. 2012b) at the E3 region into Escherichia coli BJ5183 for homologous recombination. All oncolytic Ads were propagated in HEK293 cells and purified by CsCl gradient centrifugation. The number of viral particle (VP) was measured from optical density at 260 nm (OD_260_), for which an absorbance value of 1 is equivalent to 1.1 × 10^12^ VP/mL (Choi et al. 2012a; Zhang et al. 2011).

### Animal study

Six- to eight-week-old male C57BL/6 mice and Syrian hamster were purchased from Orient Bio Inc. (Sungnam, Korea) and maintained in a laminar air-flow cabinet under a pathogen-free environment. All facilities were approved by the Association for Assessment and Accreditation of Laboratory Animal Care. All animal experiments were approved by the Hanyang University Institutional Animal Care and Use Committee (IACUC), under approval number 2021-0193 A.

B16-F10 (5 × 10^5^) or HaP-T1 (3 × 10^6^) cells were injected subcutaneously into the right abdomen of 6- to 7-week-old male C57BL/6 mice and Syrian hamsters, respectively. αCTLA-4 (InVivoMAb anti-mouse CTLA-4 clone 9H10), αPD-1 (InVivoMAb anti-mouse PD-1 clone RPM1-14), or αPD-L1 (InVivoMAb anti-mouse PD-L1 clone 10 F.9G2) were obtained from BioXCell (Lebanon, NH). When the average tumor volume reached 100 mm^3^, mice or hamsters were assigned to different treatment groups then intratumorally injected with oncolytic Ad (RdB/IL12/GMCSF, RdB/IL12/shVEGF, RdB/IL12/DCN, RdB/IL12/GMCSF-TK, or RdB/IL12/GMCSF-RLX) suspended in 50 μL PBS (designated as Day 1). The date of first treatment administration was designated as day 1. Syrian hamsters were injected on days 1 and 3, whereas the mice were treated on days 1, 3, and 5. The ICIs (αCTLA-4, αPD-1, or αPD-L1) were intraperitoneally administered (200 μg/dose in mice; 700 μg/dose in Syrian hamsters) on days 3, 6, and 9. For tumor rechallenge protocol, mice and hamsters with completely regressed primary tumors on day 50 (mouse) or 49 (Syrian hamster) were rechallenged by subcutaneously injecting 5 × 10^5^ B16-F10 or 3 × 10^6^ HaP-T1 cells into the left abdominal region of the mice or Syrian hamsters, respectively. Tumor growth was monitored every day by measuring two perpendicular tumor diameters using electronic calipers (GPM, Gneupel, Switzerland). Tumor volume was calculated using the following formula: volume = 0.523*L*(*W*)^2^ where *L* is length and *W* is width.

To evaluate the efficacy of combination therapy against recurrent tumors, B16-F10 tumor-bearing mice were treated with RdB/IL12/GMCSF-RLX (5 × 10⁷ PFU/dose) monotherapy or in combination with αPD-1 as described above. Subsequently, all mice with complete tumor regression on day 51 were subcutaneously re-inoculated with 5 × 10^5^ B16-F10 cells on the opposite flank of the initial tumor engraftment to establish secondary (“recurrent”) tumors. When these recurrent tumors reached an average volume of approximately 100 mm^3^, a second treatment cycle was initiated (designated as day 1 of the second treatment cycle) at fivefold lower viral dose (1 × 10⁷ PFU/dose). The tumor growth was monitored for 31 days from the start of the second treatment cycle.

### Quantification of cytokine expression level in tumor tissue and Th1/Th2 ratio in splenocytes

Tumor tissues were collected from mice treated with PBS, αPD-1, RdB/IL12/GMCSF-RLX, or RdB/IL12/GMCSF-RLX combined with αPD-1 at 12 days after the initial treatment. The tumor tissues were homogenized (ART-MICCRA D-8; ART Moderne Labortechnik, Mullheim, Germany) in ice-cold radioimmunoprecipitation assay buffer (Elipis Biotech, Daejeon, Republic of Korea) with a proteinase inhibitor cocktail (Sigma, St. Louis, MO). The expression levels of IL-12, GM-CSF, and IFN-γ were measured by conventional ELISA kits (IL-12 ELISA kit: Endogen, GM-CSF ELISA kit: R&D Systems and IFN-γ ELISA kit: Endogen). In addition, Th1/Th2 type cytokine expression profiles from supernatant co-cultured with splenocytes and irradiated B16-F10 (5,000 rad) were estimated using a mouse Th1/Th2 cytometric bead array kit (BD Biosciences Pharmingen, San Diego, CA).

In Syrian hamster model, splenocytes were harvested from spleen of PBS-, αPD-1-, RdB/IL12/DCN-, or combination RdB/IL12/DCN with αPD-1-treated hamsters, minced into single-cell suspensions in PBS containing 2% of FBS. The splenocytes were then cocultured with irradiated (500 rad) HaP-T1 cells for 72 h in the presence of 6,000 U/mL of recombinant human IL-2 (Peprotech, Rocky Hill, NJ). The supernatants were collected, and a hamster IFN-γ ELISA (Catalog Number: CSB-EL011050HA) was carried out according to manufacturer’s protocol (Cusabio Biotech, Wuhan, China).

### Western blot

RLX expression was determined by western blot analysis as previously described (Lee et al. 2006). The proteins from tumor tissue extracts were separated by 10% sodium dodecyl sulfate–polyacrylamide gel electrophoresis and transferred to polyvinylidene fluoride membranes (RPN 303 F, Amersham, Arlington Heights, IL). Membranes were incubated with primary anti-RLX antibody (Ab; AbClone, Seoul, Republic of Korea) or anti-β-actin Ab (Cell Signaling Technology, Beverly, MA), then horseradish peroxidase-conjugated secondary Ab (Cell Signaling Technology).

### IFN- γ ELISpot assay using murine splenocytes

At 12 days following the initial treatment with PBS, αPD-1, RdB/IL12/GMCSF-RLX, or RdB/IL12/GMCSF-RLX combined with αPD-1, spleens were obtained aseptically from B16-F10 tumor-bearing mice, and unicellular splenocytes were prepared as described previously (Lee et al. 2006). Briefly, the splenocytes were co-cultured with irradiated (5,000 rad) B16-F10 tumor cells for 18 h. in the presence of recombinant mouse IL-2 (100 U/mL; R&D Systems). An IFN-γ ELISpot assay (BD Biosciences) was then carried out as described previously (Lee et al. 2006). Spots were measured using a computer-based immunospot system (AID ELISpot Reader System version 3.4; Autoimmun Diagnostika GmbH, Strassberg, Germany).

### Thymus area measurement

Thymuses were collected from B16-F10 tumor-bearing mice treated with PBS, αPD-1, RdB/IL12/GMCSF-RLX, or RdB/IL12/GMCSF-RLX combined with αPD-1 at 12 days after the initial treatment. All thymuses were immediately placed in RMPI 1640 medium (Gibco, Grand Island, NY) and photographed. The area of each thymus was determined using ImageJ software (National Institutes of Health, Bethesda, MD).

### Fluorescence-activated cell sorting (FACS) analysis of the splenocytes

For the assessment of memory T cell population phenotype, the spleens of B16-F10 tumor-bearing mice were harvested 12 days following the initial treatment of PBS, αPD-1 (200 μg on day 3, 6, and 9), RdB/IL12/GMCSF-RLX (5 × 10^7^ PFU on days 1, 3, and 5), or αPD-1 plus RdB/IL12/GMCSF-RLX 3. The spleens were minced into single-cell suspensions in PBS plus 2% FBS and treated with EDTA-ammonium chloride solution (0.15 M NH_4_Cl, 1 mM KHCO_3_ and 0.1 mM EDTA) to remove red blood cells. For assessment of the memory T-cell population, splenocytes were stained with PerCP-CY5.5-conjugated anti-CD4 or anti-CD8 (BD Biosciences), phycoerythrin-conjugated anti-CD44 (eBioscience, San Diego, CA), fluorescein isothiocyanate-conjugated anti-CD62L (eBioscience), or APC-conjugated anti-CD127 (eBioscience) monoclonal Abs. The cells were then fixed with 1% paraformaldehyde in PBS then analyzed using a BD Biosciences BD-LSR II Analytic Flow Cytometer and FACSDiva software (BD Biosciences).

### Histology and immunohistochemistry

Representative sections were stained with haematoxylin and eosin, and then examined by light microscopy. HaP-T1 tumor sections were incubated at 4 °C overnight with anti-collagen I (Abcam, Ltd., Cambridge, UK) and anti-collagen III (Sigma) primary antibody and then incubated at room temperature for 20 min with the Dako EnvisionTM Kit (Dako, Glostrup, Denmark) as secondary antibody. Diaminobenzidine/hydrogen peroxidase (Dako) was used as the chromogen substrate. All slides were counterstained with Meyer’s haematoxylin (Sigma). The expression levels of collagen I and III were semi-quantitatively analyzed using ImageJ software. The results are expressed as the mean optical density from six different digital images.

### Statistical analysis

All these analyses were performed by two-tailed Student t test or one-way ANOVA (SPSS 13.0 software; SPSS, Chicago, IL). Data are expressed as mean ± standard error (SE). *P* values less than 0.05 were assumed to denote statistical significance.

## Supplementary Information

Supplementary Material 1.

## Data Availability

The data that support the findings of this study are available from the corresponding author, Chae-Ok Yun, upon reasonable request.
