# Supplementary material for: Enhanced potency of immune checkpoint inhibitors against poorly immunological solid tumors by immune stimulatory oncolytic adenoviruses-mediated remodeling of the tumor microenvironment
Source: Mol Med. 2025 May 7;31:175. doi: 10.1186/s10020-025-01223-4 (PMC12057182; doi:10.1186/s10020-025-01223-4)
Supplement: Supplementary file 1 — Supplementary Material 1. [file 10020_2025_1223_MOESM1_ESM.docx]

**Enhanced potency of immune checkpoint inhibitors against poorly immunological solid tumors by immune stimulatory oncolytic adenoviruses-mediated remodeling of the tumor microenvironment**

*Hyo Min Ahn^4^, Bo-Kyeong Jung^4^, JinWoo Hong^4^, Dayoung Hong^1^, A-Rum Yoon^1,2,3^* Chae-Ok Yun^1,2,3,4^**

^1^Department of Bioengineering, College of Engineering, Hanyang University, Seoul, Republic of Korea, ^2^Institute of Nano Science and Technology (INST), Hanyang University, Seoul, Republic of Korea, ^3^ Hanyang Institute of Bioscience and Biotechnology (HY-IBB), Hanyang University, Seoul, Republic of Korea, ^4^GeneMedicine Co., Ltd., 222 Wangsimni-ro, Seongdong-gu, Seoul, Republic of Korea

Keywords: c*ancer immunogene therapy, oncolytic adenovirus, programmed cell death protein 1, programmed death-ligand 1, cytotoxic T lymphocyte-associated protein 4, combination therapy*

* Correspondence should be addressed to A-R. Yoon ([ayoon@hanyang.ac.kr](mailto:ayoon@hanyang.ac.kr)) or C-O. Yun ([chaeok@hanyang.ac.kr](mailto:chaeok@hanyang.ac.kr)).

**Supplementary Figure Legends**

**Supplementary Figure**. Representative flow cytometry plots and quantification of activated T cells (CD3⁺CD4⁺CD69⁺ and CD3⁺CD8⁺CD69⁺) in the spleens of B16-F10 tumor-bearing mice treated with PBS, αPD-1 (200 μg on days 3, 6, and 9), RdB/IL12/GMCSF-RLX (5 × 10⁷ PFU on days 1, 3, and 5), or the combination therapy. Spleens were harvested on day 12 post-treatment and processed into single-cell suspensions. Following staining with fluorochrome-conjugated antibodies, activated T cell subsets were analyzed by flow cytometry. Quantification showed a significant increase in CD4⁺CD69⁺ cells in the combination group compared to PBS, while CD8⁺CD69⁺ cells exhibited an upward trend.

Representative plots and quantification of IFN-γ–producing T cells (CD3⁺CD4⁺IFN-γ⁺ and CD3⁺CD8⁺IFN-γ⁺) from splenocytes stimulated with PMA and ionomycin in the presence of Golgi transport inhibitors (Brefeldin A and Monensin) for 4 hours. A marked increase in CD4⁺IFN-γ⁺ (Th1) cells was observed in the RdB/IL12/GMCSF-RLX + αPD-1 group compared to PBS and αPD-1 groups, whereas CD8⁺IFN-γ⁺ cells showed a similar increasing trend, though without statistical significance.

Flow cytometric analysis of regulatory T cells (CD4⁺CD25⁺Foxp3⁺) and NK cells (CD3⁺NK1.1⁺CD69⁺ and CD3⁻NK1.1⁺CD69⁺) revealed no statistically significant differences among the treatment groups.


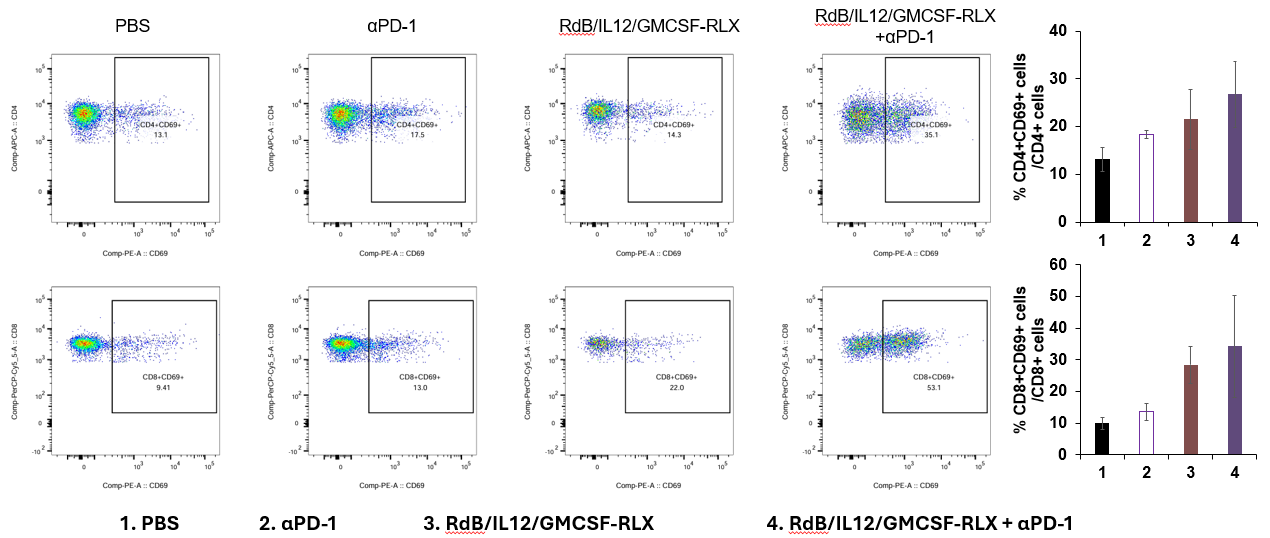


**
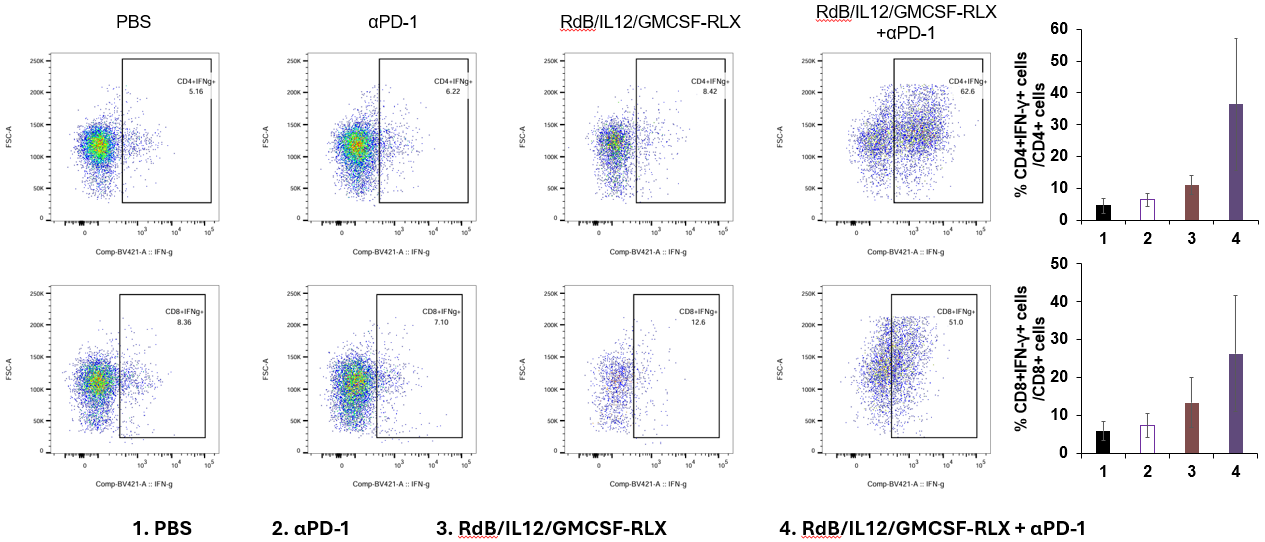
**

**
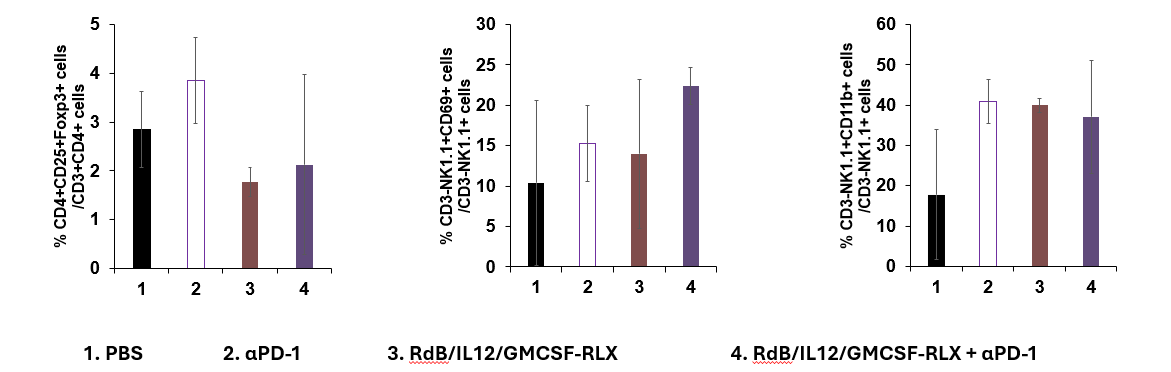
**

**Materials & Methods**

***Flow Cytometry Analysis of Immune Cell Populations***

To evaluate immune cell subset activation following treatment, spleens were harvested from B16-F10 tumor-bearing C57BL/6 mice on day 12 after initial administration of PBS, αPD-1 (200 μg on days 3, 6, and 9), RdB/IL12/GMCSF-RLX (5 × 10⁷ PFU on days 1, 3, and 5), or the combination of αPD-1 and RdB/IL12/GMCSF-RLX. Spleens were mechanically dissociated into single-cell suspensions in PBS supplemented with 2% FBS, and red blood cells were lysed using ammonium-chloride-potassium (ACK) lysis buffer (0.15 M NH₄Cl, 1 mM KHCO₃, 0.1 mM EDTA).

For detection of intracellular cytokine expression, splenocytes were stimulated with PMA (50 ng/mL), ionomycin (500 ng/mL), brefeldin A (10 μg/mL), and monensin (2 μM) in RPMI 1640 for 4 hours at 37°C. Live/dead discrimination was performed using a fixable viability dye (Invitrogen), followed by surface and intracellular staining with fluorochrome-conjugated monoclonal antibodies.

Activated T cell subsets were stained with antibodies against CD3 (FITC), CD4 (APC), CD8 (PerCP-Cy5.5), CD69 (PE), and IFN-γ (eFluor 450). For NK cell analysis, cells were stained with CD3 (FITC), NK1.1 (APC), CD11b (APC-Cy7), and CD69 (PE). Regulatory T cells were identified using antibodies against CD3 (FITC), CD4 (BB700), CD25 (PE-Cy7), CD45 (APC), and intracellular Foxp3 (PE). Intracellular staining was performed using the IC Fixation Buffer and Permeabilization Buffer (Invitrogen) or Foxp3/Transcription Factor Staining Kit (Invitrogen), according to the manufacturer's protocols.

Flow cytometric acquisition was performed using a FACSymphony A3 Cell Analyzer (BD Biosciences), and data were analyzed with FlowJo software (BD Biosciences).

***Gating Strategy for Activated T Cell Population in the Spleen***

Single cells were first gated based on size to exclude doublets, and dead cells were removed using a viability dye. CD3⁺ T cells were subsequently gated and further subdivided into CD4⁺ and CD8⁺ subsets. Within each subset, CD69 expression and intracellular IFN-γ production were analyzed to assess activation status.

**
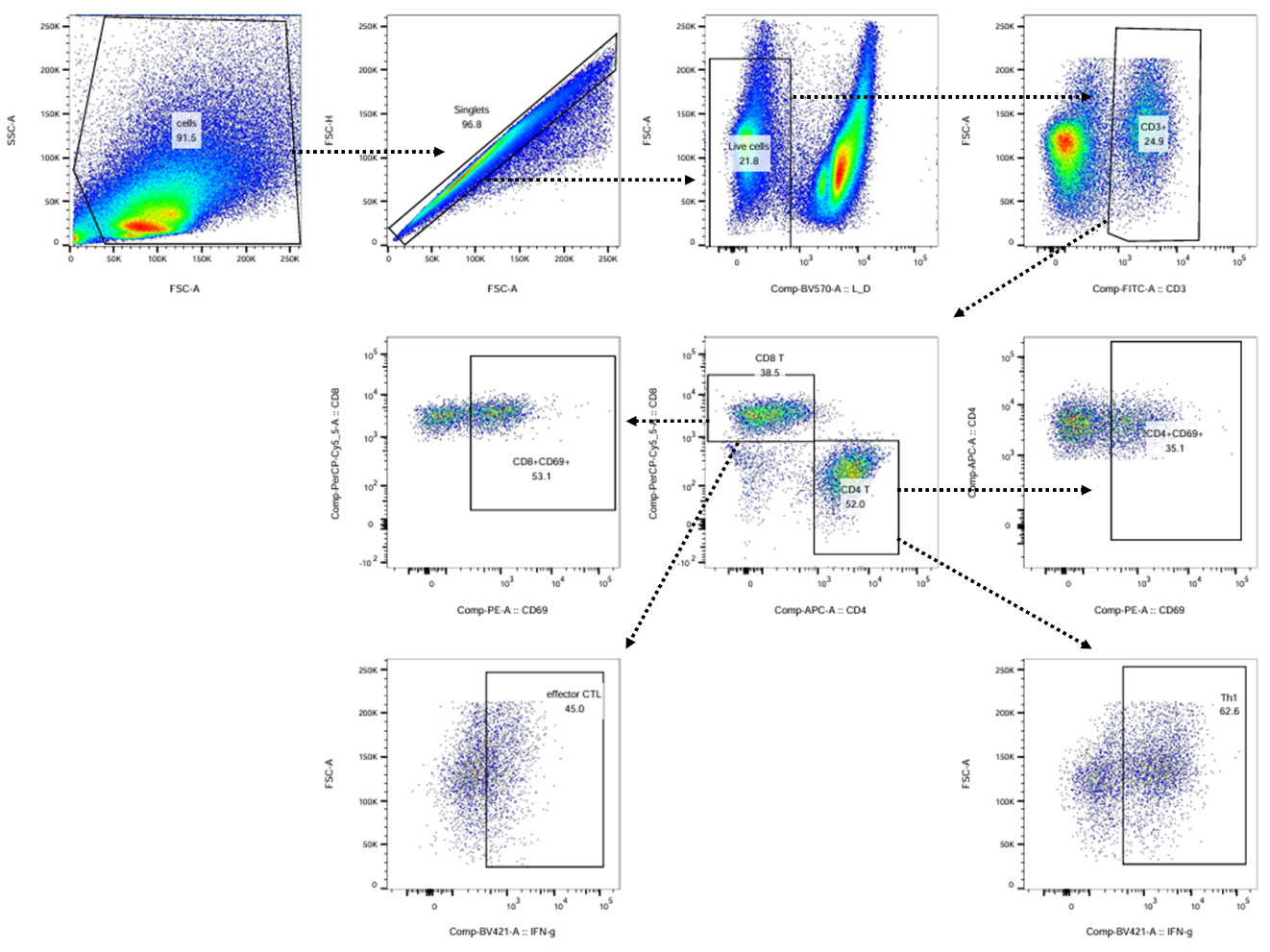
**

***Gating strategy for regulatory T cell population in spleen***

Single cells were gated based on size to exclude doublets, and dead cells were removed using a viability dye. Lymphocytes were gated based on CD45 expression, followed by identification of CD3⁺CD25^high cells. Within this population, Foxp3⁺ cells were analyzed as regulatory T cells.

**
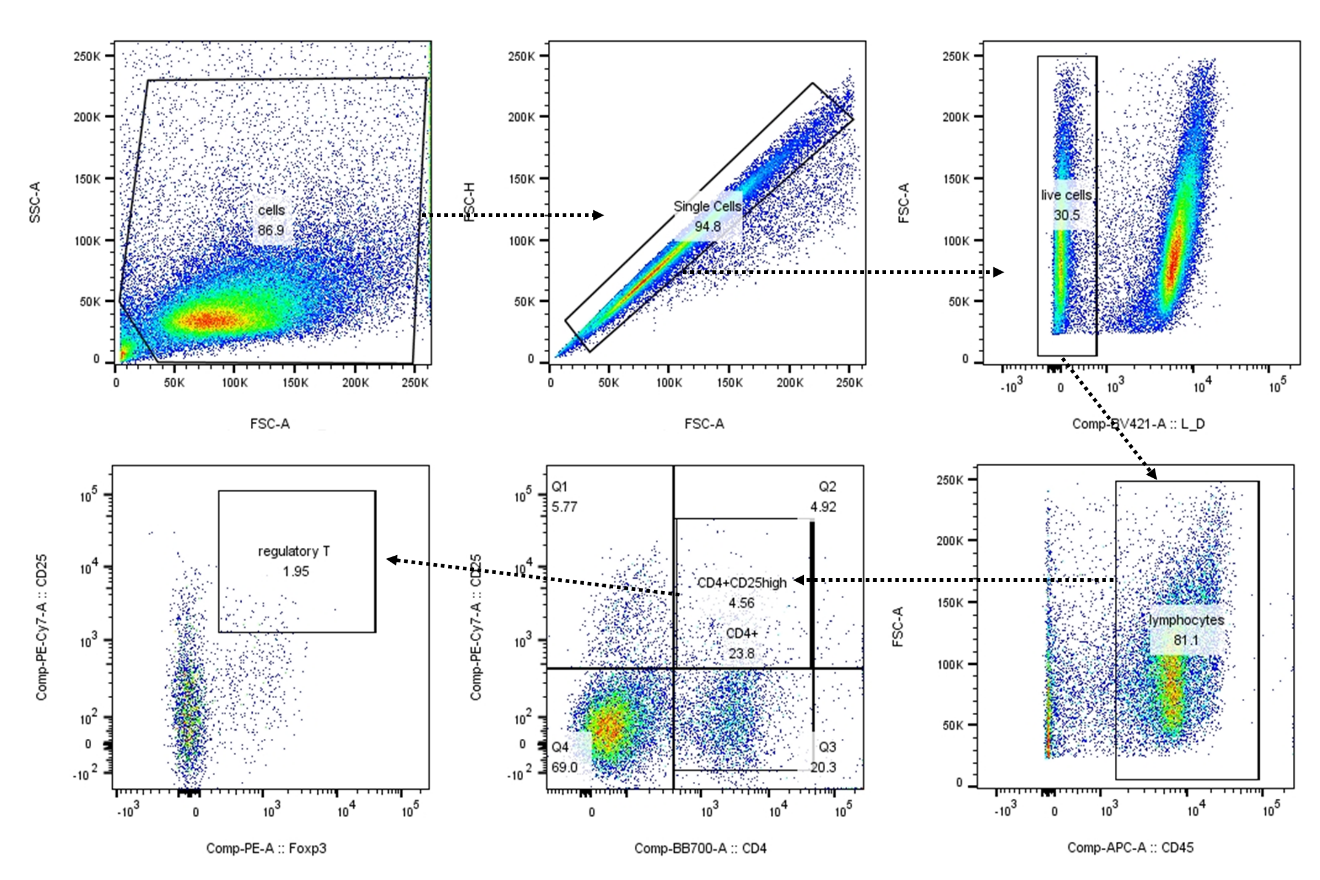
**

***Gating strategy for NK cell population in spleen***

Single cells were gated based on size to exclude doublets, and dead cells were removed using a viability dye. CD3⁻NK1.1⁺ cells were gated as the NK cell subset. Within this population, CD69⁺ cells were analyzed as activated NK cells, and CD11b⁺ cells were analyzed to assess maturation status.

**
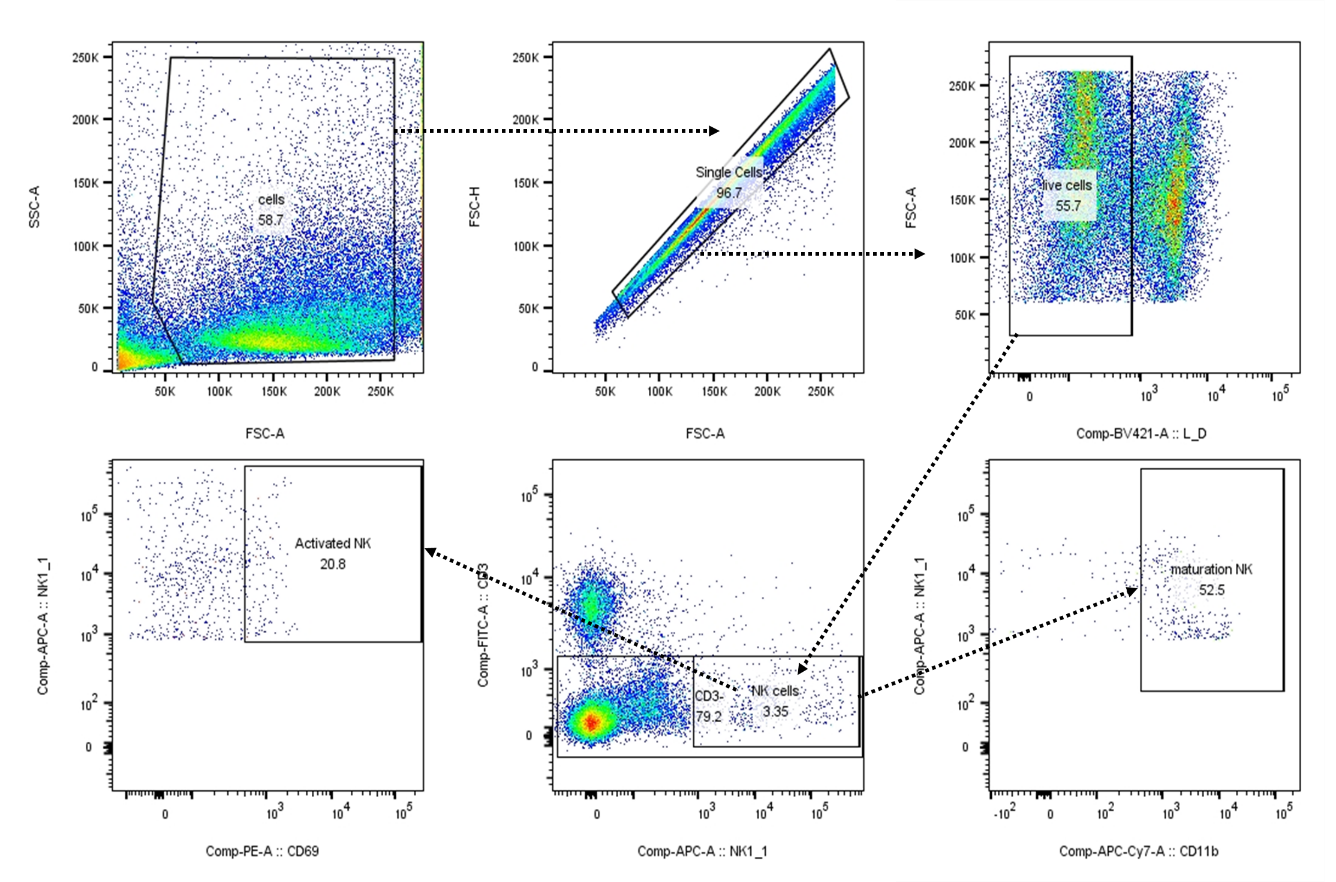
**
